# Cutaneous Disease as the First Manifestation of Cystic Echinococcosis

**DOI:** 10.4269/ajtmh.15-0855

**Published:** 2016-08-03

**Authors:** Virginia Velasco-Tirado, Manuela Yuste-Chaves, Moncef Belhassen-García

**Affiliations:** ^1^ Servicio de Dermatologia, Complejo Asistencial Universitario de Salamanca (CAUSA), Centro de Investigación de Enfermedades Tropicales de la Universidad de Salamanca (CIETUS), Instituto de Investigación Biomedica de Salamanca (IBSAL), Universidad de Salamanca; ^2^ Salamanca, Spain; Servicio de Medicina Interna, Seccion de Enfermedades Infecciosas, Complejo Asistencial Universitario de Salamanca (CAUSA), Centro de Investigación de Enfermedades Tropicales de la Universidad de Salamanca (CIETUS), Instituto de Investigación Biomedica de Salamanca (IBSAL), Universidad de Salamanca, Salamanca, Spain

**Figure 1.**
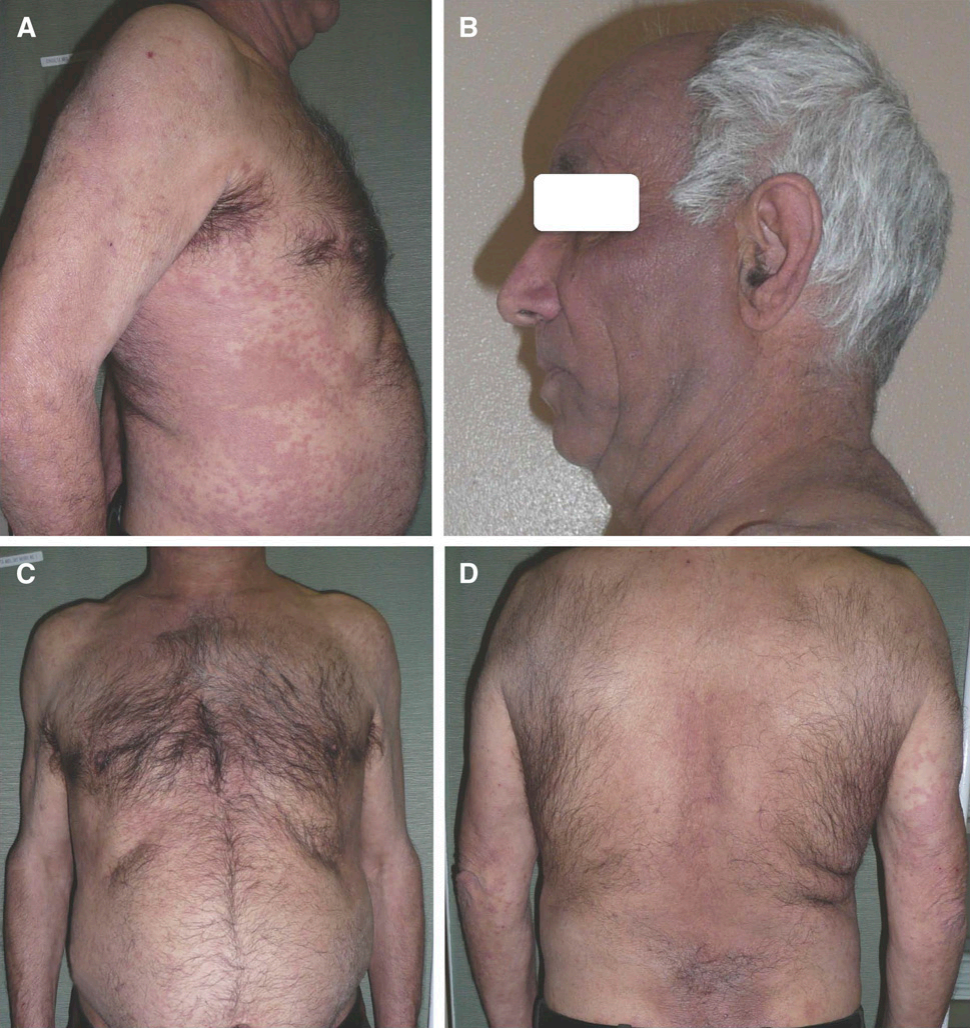
(**A**, **C**, **D**) Xerosis, erythematous scaly skin areas with lichenification and hyperpigmentation of the trunk and upper limbs. (**B**) Greyish hyperpigmentation and severe lichenification and infiltration on the patient's face.

A 61-year-old man from a rural area (Salamanca, Spain), who had contact with dogs, was admitted with generalized itching for 4 years. He was treated with oral antihistamines. A physical examination revealed greyish hyperpigmentation and severe lichenification and infiltration on the face, without mucosal pigmentation. His trunk and limbs showed xerosis, erythematous scaly skin areas with lichenification and hyperpigmentation (Figure 1

).

**Figure 2.**
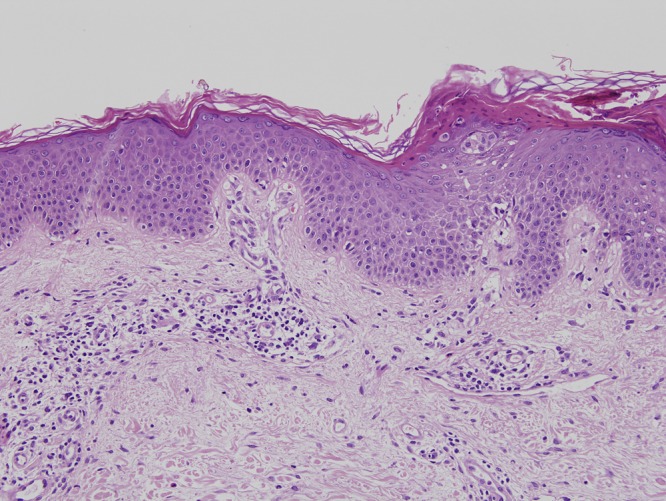
Perivascular spongiotic dermatitis with eosinophilic infiltrate in the histopathological examinations of skin biopsy (H-E × 10).

Increased levels of IgE of 2,864 UI/L (0–114 IU/L), but no eosinophilia, were detected. Skin biopsy revealed perivascular spongiotic dermatitis with eosinophilic infiltrate, congruent with eczema (Figure 2

). Allergic and photoallergic contact dermatitis and aeroallergen sensitization were ruled out. Bronchial hyperresponsiveness was determined and the patient was treated with salbutamol inhalation.

**Figure 3.**
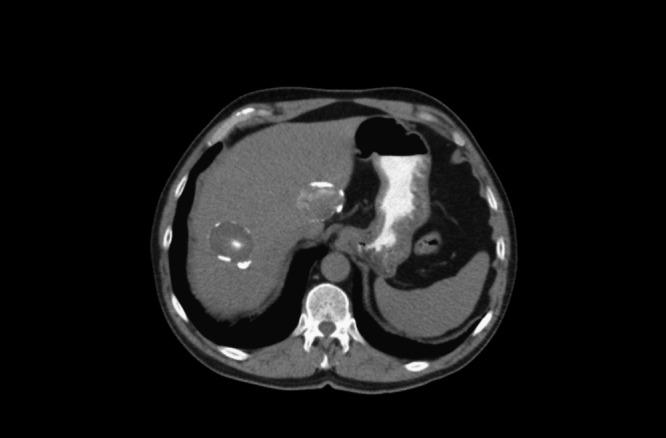
Computerized tomography showing hepatic hydatid cysts.

**Figure 4.**
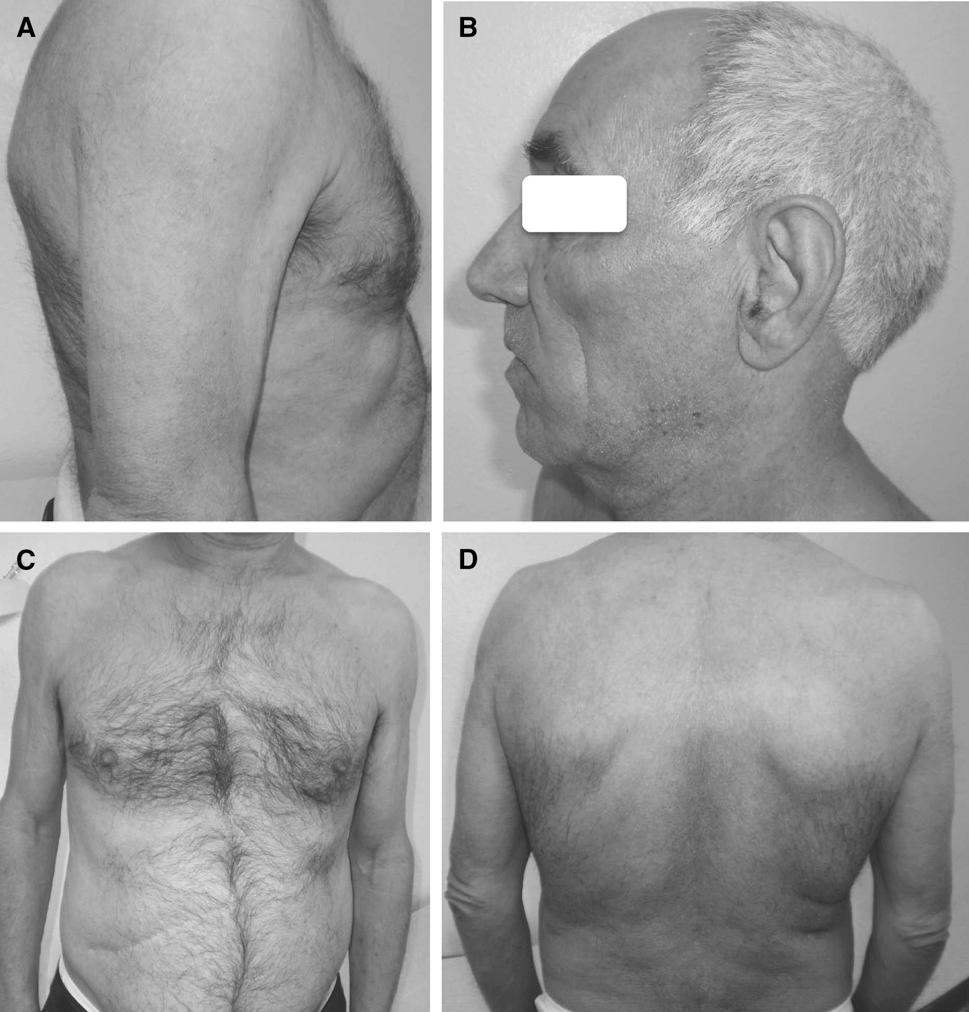
(**A**–**D**) Clear improvement of skin lesions, with (**D**) hyperpigmentation only in the trunk.

After a diagnosis of generalized eczema, he was managed with topical propionate of clobetasol and topical tacrolimus, oral ebastine, and oral prednisone in a tapering regimen during flares. Skin lesions worsened with bronchial reactivity 4 years later. IgE > 5,000 UI/L and eosinophilia of 900/μL (7.19%) were detected. Chest X-ray was normal. Antibodies against hepatitis B virus, hepatitis C virus, syphilis, *Trichinella* sp., *Toxoplasma gondii*, *Strongyloides* sp., *Fasciola hepatica*, *Taenia solium*, and parasites in stool (three serial samples) were negative. IgG results for hydatic disease were repeatedly negative, but specific *Echinococcus granulosus* IgE was detected (3.13 kUA/L) (negative < 0.35 kUA/L, ImmunoCAP system, Phadia, Uppsala, Sweden). Abdominal computerized tomography showed three focal lesions that were consistent with hepatic hydatid cysts: the first cyst was localized in segment I of 24 × 21 × 18 cm (stage cystic echinococcosis [CE] 5), the second cyst in segment II of 48 × 31 × 36 cm (stage CE3), and the third cyst in segment VII of 45 × 34 × 34 cm (stage CE3) (Figure 3

). Albendazole (400 mg twice a day) and praziquantel (1,200 mg twice a day) were administered and surgery was subsequently performed. Removal of cysts in segment I, II, and VII was done. Histopathological examination confirmed infection by *E. granulosus*. Treatment with only albendazole was continued because of digestive intolerance from praziquantel. The patient improved symptomatically and with regard to the skin lesions (Figure 4

). All treatments (topical, oral, and inhaled) were stopped after 18 months.

In dermatology, increased levels of IgE and eosinophilia are commonly related to atopy, but other entities with skin manifestations, mainly neoplasms and infectious diseases, should also be considered. CE is a chronic, complex, and neglected zoonotic disease, and it remains an important health problem in many areas of the world.^1^ In humans, it may result in a wide spectrum of clinical manifestations, ranging from asymptomatic infection to severe and even fatal disease.^2^ CE typically grows slowly and may long remain clinically silent. Common serodiagnotis techniques may produce a high percentage of false-negative results, and thus CE diagnosis can be difficult.^3^ *Echinococcus granulosus* infection may produce different cutaneous manifestations, some of which are due to mechanical complication, such as skin fistulae,^4^ and others are due to anaphylactoid reactions, such as acute or chronic urticaria and flushing.^5^ It is assumed that these former symptoms may be caused by a partial rupture of the cyst with microscopic drainage. We propose that this continuous antigenic trigger and repeated scratching could potentially result in clinical manifestations in our patient, which were resolved using antiparasitic treatment. We have not found any previously described association between the skin alterations in our patient and hydatid disease. In conclusion, we highlight that skin manifestations may be a clue in the diagnosis of potentially severe infectious diseases, and we should include CE in the differential diagnosis of generalized eczema.
